# Supplementary material for: Recovering high-resolution information using energy filtering in MicroED
Source: bioRxiv. 2025 Mar 26:2025.03.26.645403. Preprint. [Version 1] doi: 10.1101/2025.03.26.645403 (PMC11974828; doi:10.1101/2025.03.26.645403)
Supplement: Supplement 1 [file NIHPP2025.03.26.645403v1-supplement-1.pdf]

bioRxiv preprint doi: https://doi.org/10.1101/2025.03.26.645403; this version posted March 26, 2025. The copyright holder for this preprint (which was not certified by peer review) is the author/funder, who has granted bioRxiv a license to display the preprint in perpetuity. It is made available under aCC-BY 4.0 International license.

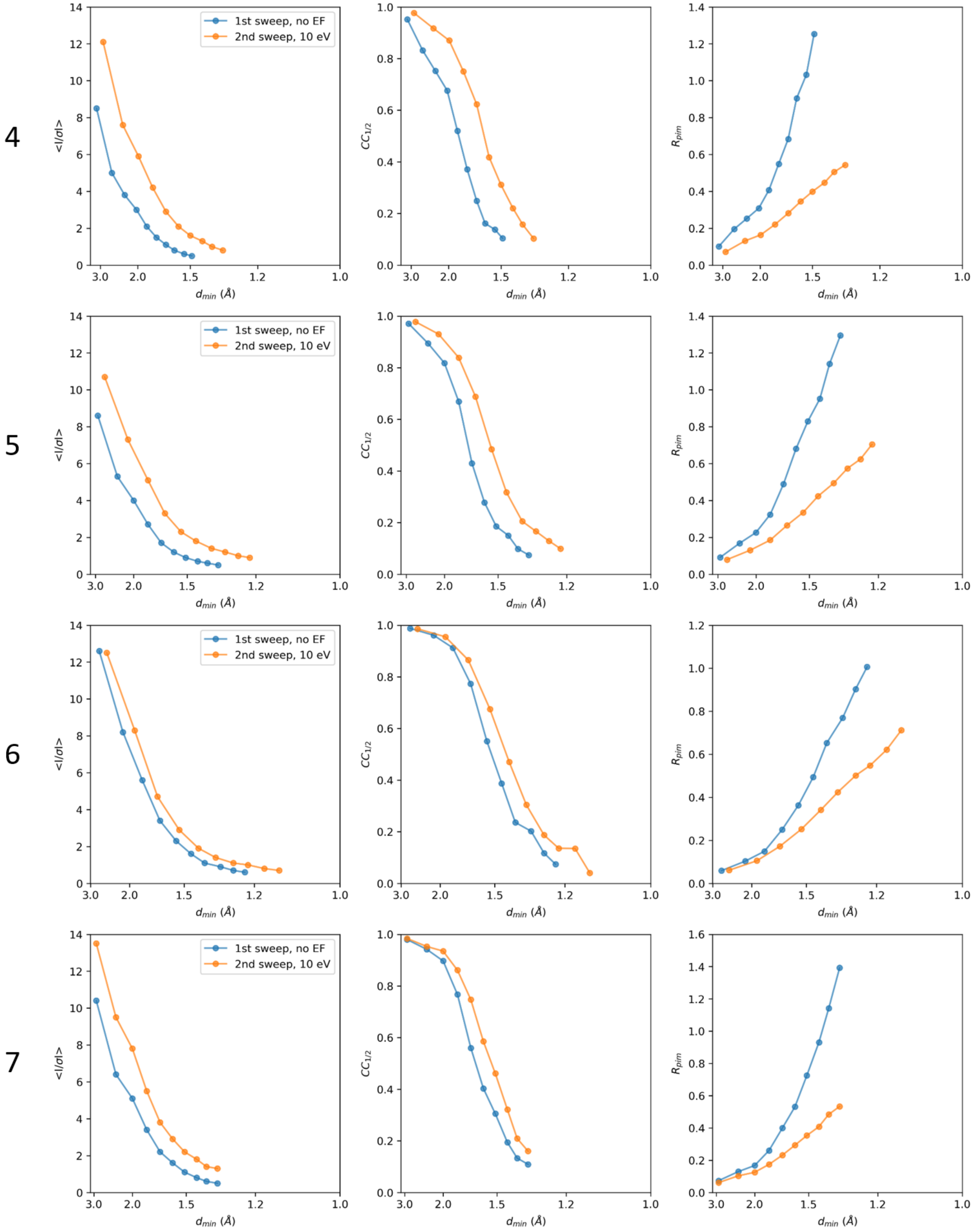

Figure S1. Intensity statistics for four MicroED data collection series. For each series, two datasets were collected from the same lamellae where the first pass did not use energy filtering (blue), whereas the second pass had the 10 eV energy filter slit inserted (orange). For each lamella, the crystallographic quality indicators mean $I/\sigma I$, $CC_{1/2}$, and R_{pim} are plotted as function of the resolution. Individual datasets were truncated at a $CC_{1/2}$ that was still significant at the 0.1% level in the highest resolution shell.

bioRxiv preprint doi: https://doi.org/10.1101/2025.03.26.645403; this version posted March 26, 2025. The copyright holder for this preprint (which was not certified by peer review) is the author/funder, who has granted bioRxiv a license to display the preprint in perpetuity. It is made available under aCC-BY 4.0 International license.

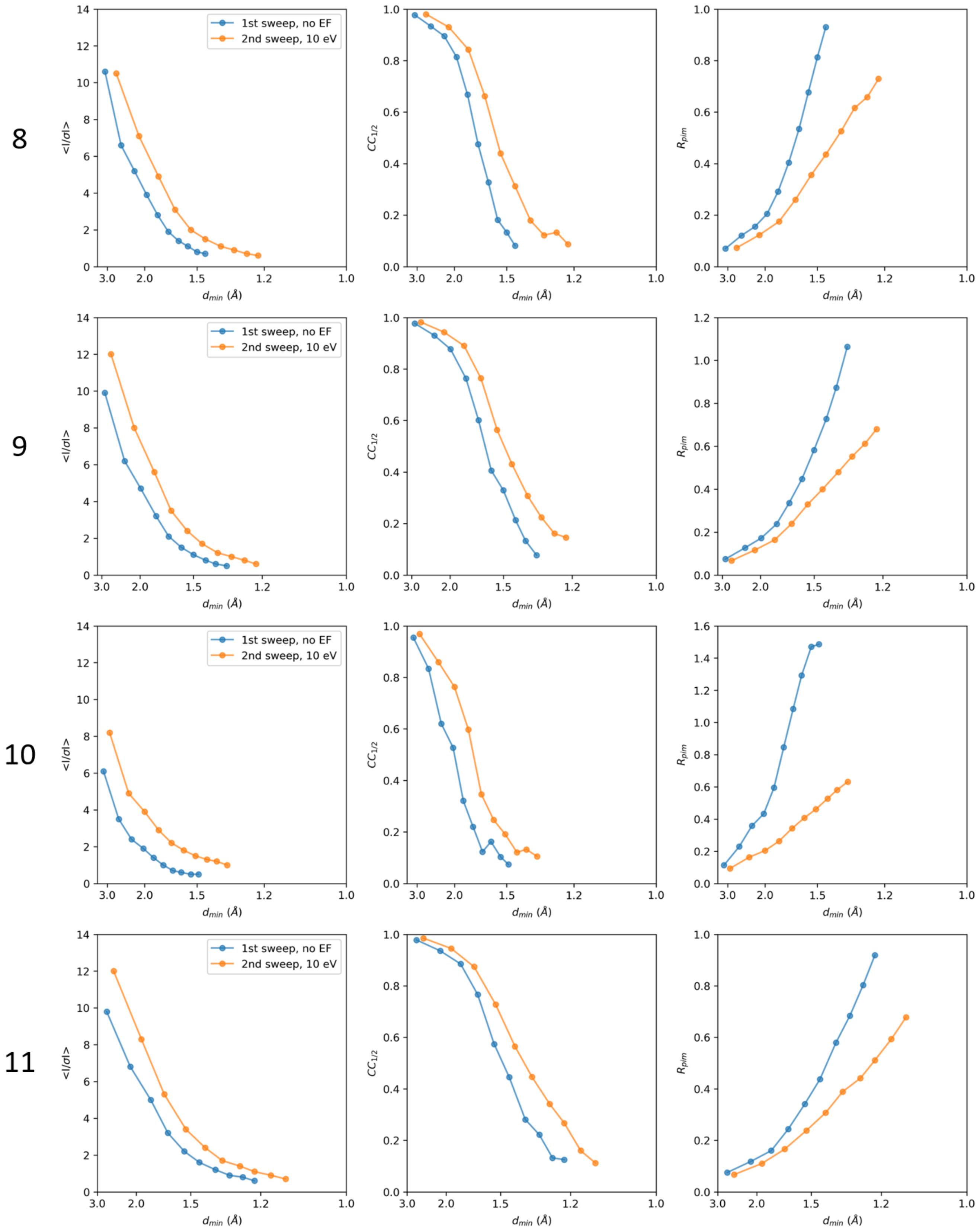

Figure S2. Intensity statistics for four MicroED data collection series. For each series, two datasets were collected from the same lamellae where the first pass did not use energy filtering (blue), whereas the second pass had the 10 eV energy filter slit inserted (orange). For each lamella, the crystallographic quality indicators mean $I/\sigma I$, $CC_{1/2}$, and R_{pim} are plotted as function of the resolution. Individual datasets were truncated at a $CC_{1/2}$ that was still significant at the 0.1% level in the highest resolution shell.
